# Detecting Honey Adulteration: Advanced Approach Using UF-GC Coupled with Machine Learning

**DOI:** 10.3390/s24237481

**Published:** 2024-11-23

**Authors:** Irene Punta-Sánchez, Tomasz Dymerski, José Luis P. Calle, Ana Ruiz-Rodríguez, Marta Ferreiro-González, Miguel Palma

**Affiliations:** 1 Department of Analytical Chemistry, Faculty of Sciences, University of Cadiz, Agrifood Campus of International Excellence (ceiA3), IVAGRO, 11510 Puerto Real, Spain; irene.punta@uca.es (I.P.-S.); joseluis.perezcalle@uca.es (J.L.P.C.); ana.ruiz@uca.es (A.R.-R.); miguel.palma@uca.es (M.P.); 2 Department of Analytical Chemistry, Faculty of Chemistry, Gdańsk University of Technology, 11/12 G, Narutowicza Str., 80-233 Gdansk, Poland; tomasz.dymerski@pg.edu.pl

**Keywords:** honey, adulteration, ultra-fast gas chromatography, machine learning, regression, classification, food control, volatile compounds

## Abstract

This article introduces a novel approach to detecting honey adulteration by combining ultra-fast gas chromatography (UF-GC) with advanced machine learning techniques. Machine learning models, particularly support vector regression (SVR) and least absolute shrinkage and selection operator (LASSO), were applied to predict adulteration in orange blossom (OB) and sunflower (SF) honeys. The SVR model achieved R^2^ values above 0.90 for combined honey types. Treating OB and SF honeys separately resulted in a significant accuracy improvement, with R^2^ values exceeding 0.99. LASSO proved especially effective when honey types were treated individually. The integration of UF-GC with machine learning not only provides a reliable method for detecting honey adulteration, but also sets a precedent for future research in the application of this technique to other food products, potentially enhancing food authenticity across the industry.

## 1. Introduction

Honey is a natural sweetener produced by *Apis mellifera* L. bees from the nectar of plants or from secretions of living parts of plants or excretions of plant-sucking insects on the living parts of plants [1]. The composition and properties of honey depend on the botanical origin of the source of nectars or secretions, climatic conditions, environmental factors, and bee farming practices. Honey can be classified into two categories depending on the secretions of plants used for their synthesis: blossom honey made from the nectar of flowers, and honeydew honey made from secretions of all living parts of plants other than flowers or excretions of insects [2,3].

Monofloral honey is a type of honey produced from a single botanical source holding distinctive organoleptic properties. [4]. Monofloral honey is generally considered to be more valuable than multifloral honey because it is more difficult to produce, and it has a unique flavor profile that is specific to the flower or plant from which it was derived. In the case of orange blossom (OB) and sunflower (SF) honeys, their distinct flavors and aromas make them highly desirable. OB honey has a delicate floral aroma and a sweet citrus taste [5], while sunflower honey has a mild and pleasant flavor with a light floral aroma.

According to the European Union Council Directive 2001/110/EC [6] and FAO/WHO Codex Alimentarius [1], honey is defined as a natural sweet product, so that the addition of any foreign materials, and falsely declaring the botanical or geographical origins, are all considered fraudulent practices. The addition of cheap sweeteners or lower quality honey is a common form of honey adulteration, which aims to increase the volume and sweetness of the product while reducing production costs [7,8]. However, this practice can also compromise the quality and safety of the honey. Additionally, adulterated honey may have a different composition, taste, and aroma than pure honey [9,10].

To ensure the quality and safety of honey, various national and international organizations have established standards and guidelines for honey production, processing, and labeling. The Codex Standard for Honey is a globally recognized standard that sets the minimum quality and purity criteria for honey, including its moisture content, sugar composition, and absence of additives and contaminants [1,11].

In recent years, several techniques have been developed to detect honey adulteration, including stable carbon isotope ratio analysis (SCIRA) [12]; gas chromatography (GC) [13,14]; ion mobility spectrometry (IMS) [15]; nuclear magnetic resonance (NMR) [16]; Fourier transform infrared spectroscopy with attenuated total reflectance accessory (FTIR-ATR) [17]; UV–Visible (UV-Vis) [18], near-infrared (NIR) [19], Raman spectroscopy [20], and thermographic images [21]; and biosensor technology [22]. These techniques have been employed to detect the most common honey adulterants such as refined cane sugar, beet sugar, or corn syrup. However, they have different strengths and limitations, and the adulteration of high-cost honeys, including monofloral honeys, with low-cost honeys is not easily detected using these techniques [23] (Table 1). SCIRA analyzes the ratio of stable carbon isotopes and is limited in its ability to detect other types of adulteration. GC separates and analyzes volatile honey components but may have limited sensitivity for certain adulterants. IMS also identifies volatile compounds but also has limitations in sensitivity. NMR provides detailed molecular information but requires expensive equipment and complex data interpretation. FTIR-ATR analyzes the infrared spectrum and may have limited sensitivity for certain adulterants. UV-Vis measures light absorption but has limited specificity. NIR analyzes the near-infrared region and most organic compounds in food samples contribute to the absorption in the NIR region. Therefore, as specific spectroscopic regions with individual contributions from specific compounds are usually not available, samples previously analyzed with a reference method should be used to prepare calibration curves while developing analytical methods based on NIR spectroscopy, instead of the regular calibration procedures with standards. Raman spectroscopy identifies chemical bonds and structures but may require complex data analysis. Thermographic images measure temperature variations but have limited specificity. Biosensor technology detects specific adulterants but is limited to targeted adulterants and may require optimization.

Ultra-fast gas chromatography (UF-GC) is a powerful analytical tool that allows the rapid and sensitive analysis of individual volatile compounds in complex mixtures such as honey. Unlike traditional gas chromatography, which can be time-consuming and costly, UF-GC completes its detection process in just a few hundred seconds, providing rapid results [24]. The most common approach in the analysis of the volatile fraction using gas chromatography is the identification and determination of target compounds. Such an approach is not possible when using ultra-fast gas chromatography due to the short length of chromatographic columns and steep temperature ramps. In these cases, a non-targeted chemical analysis approach can be used instead [25]. Machine learning (ML) algorithms have been previously employed for detection of adulterants in honey with other techniques [26]. In most cases, honey adulteration involves the addition of artificial sweeteners or syrups [17,27]. However, in this study, we focused on adulterating high-value honey with cheaper varieties. Detecting this type of honey-to-honey adulteration is more challenging and requires advanced techniques. We employed UF-GC combined with machine learning algorithms as a novel approach for developing precise and reliable models to identify adulterants in food, in this case, lower-value honey mixed into higher-value honey.

The aim of this study was to evaluate the ability of different ML algorithms in combination with UF-GC to predict the level of adulteration in orange blossom and sunflower honeys. The following algorithms were evaluated: least absolute shrinkage and selection operator (LASSO), ridge regression (RIDGE), elastic net (ENET), partial least squares (PLS), random forest (RF), and support vector regression (SVR). These ML algorithms have demonstrated successful applications in the detection and quantification of adulterations in honey using Vis-NIR [28]. This study is the first to examine and compare the performance of some ML regression techniques for the determination of honey adulteration.

## 2. Materials and Methods

### 2.1. Samples

In order to cover the widest heterogeneity, a mixture of OB honey was prepared by mixing 13 different pure OB honeys. Likewise, a mixture of SF honey was prepared by mixing 7 different pure SF honeys. The pure OB and SF honeys were provided by the Andalusian Agency for Agricultural and Fisheries Management (AGAPA) from the Andalusian Government as a part of the collection in the Wine and Food Research Institute (IVAGRO) at the University of Cádiz (Spain), code NPM21-IVAGRO. The adulterated samples were prepared by mixing each type of honey (OB and SF) with a different proportion of adulterant. A mixture in equal proportions of eucalyptus, rosemary, and thousand flower honeys were used as the adulterant. These honeys were selected as adulterants for their affordability and availability in local markets. The quantity of adulterant in the resulting adulterated samples ranged from 5% to 50%. Each type of sample was prepared in duplicate, so a total of *n* = 44 samples were prepared for analysis. The samples were labeled according to the code XY_N_M, where XY could be SF (sunflower honey) or OB (orange blossom honey), N could be 5, 10, 15, 20, 25, 30, 35, 40, 45, or 50% of adulterant, and M could be 1 or 2 (duplicated samples).

### 2.2. Honey Analysis

Honey samples were analyzed using a Heracles II ultra-fast gas chromatography system equipped with an HS100 autosampler (Alpha M.O.S., Toulouse, France). The system was equipped with two parallel 10 m columns with different polarity (MXT-5 and MXT-1701) (Restek, Bellefonte, PA, USA). The MXT-5 column features a low-polarity phase (diphenyl dimethyl polysiloxane) for separating nonpolar and moderately polar compounds, while the MXT-1701 column has a higher-polarity stationary phase (cyanopropylmethyl phenylmethyl polysiloxane) suitable for both polar and nonpolar compounds. MXT-5 is ideal for hydrocarbons and volatile compounds, while MXT-1701 is versatile and can be used for food, environmental analysis, and compounds like alcohols and esters. Each column was coupled to a micro flame-ionization detector (µFID). The carrier gas was hydrogen having 6N purity and provided by a Precision Hydrogen Trace 250 generator (Peak Scientific Instruments, Inchinnan, UK).

Honey samples weighing 1.5 g were mixed with 5 mL of a solution containing 30% salt in water. The mixture was then poured into glass vials with a headspace of 20 mL, and the vials were sealed with caps lined with a membrane made of silicon and PTFE. The sealed vials were incubated at a temperature of 40 °C for 20 min, while being stirred at 500 rpm. The injector temperature was 200 °C. The injected headspace sampling volume was 2500 µL at 250 µL/s. Analytes were trapped in a Tenax^®^ TA sorptive material at 40 °C, and were then thermally desorbed at 240 °C. The total process took 20 s.

The oven temperature was programmed to hold 40 °C during 2 s, and then increase from 40 to 270 °C at a rate of 3 °C s^−1^, with a final hold of 18 s, and the FID detector temperature was set to 270 °C. The time of data acquisition was 100 s and the sampling rate was 100 samples s^−1^. Considering the incubation time, the final time of analysis was approximately 25 min.

### 2.3. Data Analysis

Data analyses from UF-GC results were performed with RStudio (R version 4.0.5, Boston, MA, USA). The dataset was obtained by concatenating chromatographic data from both FIDs, which resulted in a two-dimension data matrix (D*_nxp_*) of D_44*x*20002_, where *p* is the number of variables (retention times), and *n* is the number of samples (adulterated and unadulterated OB and SF honey).

Several RStudio packages were used during the analysis, including *Boruta* [29] for variable selection, *factoextra* [30] for hierarchical cluster analysis, *ggplot2* [31] for graphical visualizations, and *caret* [32] for creating machine learning models for both classification and regression: LASSO [33], RIDGE [34], ENET [35], PLS [36], RF [37], SVR, and SVM [38]. A paired *t*-test was conducted to compare the predictive performance using RMSE in each pair of ML models at a 95% level of significance.

The regression models were assessed using metrics such as R-squared (R^2^), root mean square error (RMSE), and the ratio of predicted deviation (RPD). R^2^ represents the model’s goodness of fit, with higher values indicating better fits, while RMSE measures the average difference between actual and predicted values, with lower values indicating better fits. RPD was used to evaluate model effectiveness, with the following categories: excellent for RPD > 2.5, very good for RPD between 2 and 2.5, good for RPD between 1.8 and 2, moderate for RPD between 1.4 and 1.8, weak for RPD between 1 and 1.4, and very poor for RPD < 1 [27]. To evaluate the classification models’ performance, the accuracy metric was used, which was calculated as the number of accurately classified instances divided by the total number of instances.

## 3. Results

### 3.1. Exploratory Analysis

**Figure 1:**
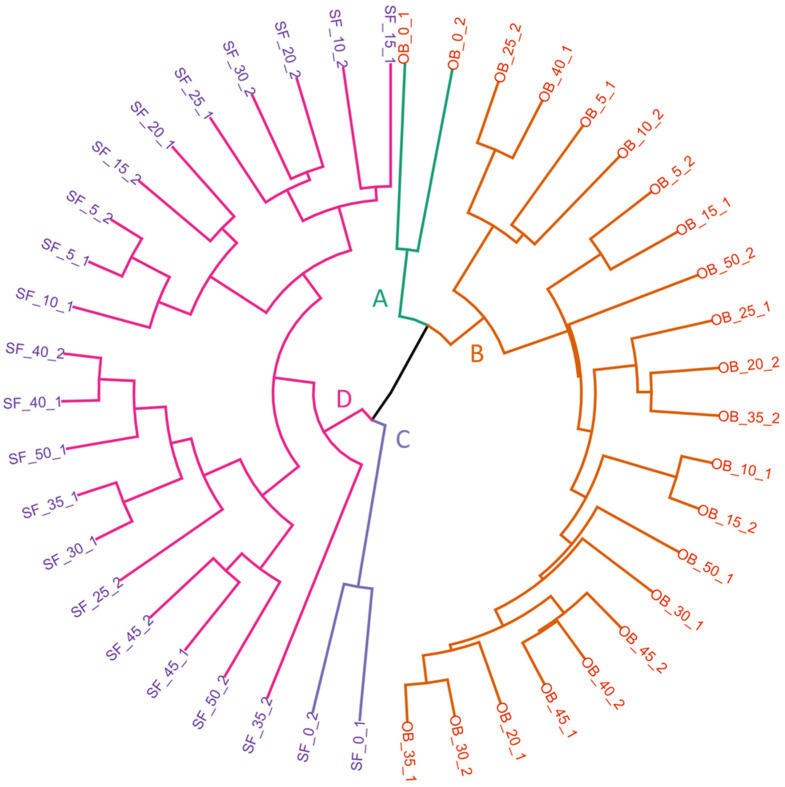
Circular dendrogram resulting from the hierarchical cluster analysis (HCA) of the dataset (D_44*x*20002_) with UF-GC; The names of the honey samples are colored according to their botanical origin: sunflower (purple) and orange blossom (orange). The four main clusters have been colored and labeled with letters A, B, C, and D. The average method with Euclidean distances was used.

First, the aim was to study whether there was any tendency of the honey samples to be classified according to the botanic origin and/or the level of adulteration based on UF-GC results. To do so, the raw data matrix containing OB and SF honey samples (D_44*x*20002_) without applying any pre-treatment was subjected to hierarchical cluster analysis (HCA). Euclidean distances were used to determine the similarity between samples. The linkage method, used to merge or split clusters based on their similarity, was selected by comparing different methods (single, full, average, Ward, and centroid). Since HCA was performed without pre-treatment and concatenating information obtained by both columns in order to understand raw tendencies, values in the linkage method ranged from 0.6216 to 0.7474. The average method obtained the best result (0.7474). The results from HCA are represented in a dendrogram in Figure 1. In general, it can be observed that samples with the same botanical origin are located in related clusters, and the level of the adulteration conditions the location in the dendrogram. The four main clusters identified are A, B, C, and D. The unadulterated samples of OB honey are grouped in cluster A, while unadulterated SF samples are grouped in cluster C. On the other hand, adulterated OB samples are grouped in cluster B and adulterated SF samples are grouped in cluster D. Adulterated SF samples showed a slight trend to cluster by their level of adulteration in subclusters. Nonetheless, this trend was less evident in the case of OB samples.

This analysis suggests that the UF-GC technique can be used to effectively distinguish between unadulterated and adulterated honey samples, as well as differentiate between different botanical origins. However, it was not possible to classify the samples according to the level of adulteration by this exploratory analysis. For this reason, and in order to develop a model capable of predicting and quantifying future samples of adulterated honey, different supervised techniques were evaluated.

### 3.2. Supervised Models for Prediction of Level of Adulterant

The application of supervised regression methods, such as LASSO, RIDGE, ENET, PLS, SVR, and RF, can help in developing models to predict the percentage of adulterant in honey samples based on the data obtained through UF-GC. Through the application of these regression methods, this study not only facilitates the detection of potential adulteration, but also identifies the most dependable method for making predictions, contributing to the quality control and authenticity of honey in the market.

To develop robust predictive models and to avoid overfitting, some data pre-treatment was performed. Boruta is a feature selection algorithm designed for identifying relevant features in high-dimensional datasets and categorizes features as “important”, “unimportant”, or “tentative”. The Boruta algorithm assesses the importance of each feature in an original dataset by comparing its score to a set of shadow attributes, representing noise, using a RF classifier. Features that are significantly more important in the original dataset, compared to the shadow dataset, are considered relevant and are retained for the model. This iterative process helps to select the most informative features, reducing the risk of overfitting and enhancing model performance. It enhances the quality of predictive models by effectively identifying the most relevant features while mitigating the risk of overfitting. It contributes to the overall reliability and accuracy of models dealing with UF-GC data and the prediction of adulterant percentages in honey samples.

In this case, the feature selection algorithm Boruta was applied to identify the most relevant features generated by combining data from both columns of UF-GC. For all the regression models, the dataset containing the concatenated information of both sensors (D_44_*x*_20002_) was randomly split into a 75% training set (*n* = 33) and a 25% test set (*n* = 11), only considering the level of adulteration (5–50%) and excluding the botanical origin. The test set contained independent samples that were not used in the model and was used for external validation to obtain an unbiased error estimate for all trained models. Then, only the training set (D_33_*x*_20002_) was pre-processed using the Boruta algorithm and 30 features were selected (D_33_*x*_30_) and employed to build the models. Parameter optimization was assessed by 5-fold cross-validation (CV) on the training set. Table 2 and Table 3 include the optimized parameters, the 5-fold CV performance of regression models, and the performance of regression models on the training and test sets, respectively. These models were applied to all honey samples, as well as specifically to OB and SF honey. Model performance was evaluated in both tables using RMSE and R^2^. RPD is also include in Table 3 to evaluate model effectiveness.

#### 3.2.1. Least Absolute Shrinkage and Selection Operator (LASSO)

LASSO is a linear regression method that performs both regularization and variable selection by applying a penalty to the regression coefficients, which shrinks some coefficients to zero and sets corresponding variables to be excluded from the model. The degree of penalty is controlled by the hyperparameter lambda (*λ*). Higher values of *λ* result in stronger regularization, meaning that more coefficients will be forced to be exactly zero. This reduces model complexity and reduces overfitting. However, it can introduce bias because it may underfit the data by setting too many coefficients to zero. Decreasing *λ* weakens the regularization effect, allowing more coefficients to remain non-zero, leading to higher model complexity, which might result in overfitting, increased variance, and reduced bias.

A predictive model was developed using regularization with an optimized *λ* value of 0.132. Lambda was optimized by a grid search method using exponential sequences from 10^−5^ to 10 every 100. The model achieved good performance, with an RMSE of 4.041 and an R^2^ of 0.937 on an independent dataset. On the training and test sets, the RMSE obtained was 3.745 and 6.335, and R^2^ values were 0.943 and 0.874, respectively. The method selected 2 out of the resulting 30 variables after applying feature selection with Boruta.

#### 3.2.2. Ridge Regression (RIDGE)

In RIDGE, the value of the hyperparameter lambda (*λ*) controls the amount of shrinkage applied to the coefficients. Larger values of lambda result in more shrinkage and smaller coefficients, but coefficients of less-important features are never reduced to 0. RIDGE reduces the magnitude of the coefficients and eliminates multicollinearity by spreading the influence of correlated features. This results in a reduction in variance, which can help with overfitting, while introducing some bias due to the coefficient shrinkage. Decreasing *λ* in RIDGE reduces the regularization effect, allowing the model to have larger coefficient values. This can lead to increased model complexity and higher variance, which might make the model more prone to overfitting and less biased.

The optimal value of lambda obtained by a grid search method using exponential sequences from 10^−5^ to 10 every 100 was in this case 10, which resulted in an RMSE of 6.353 and an R^2^ of 0.901. The RMSE and R^2^ values for the training set were 5.480 and 0.877, respectively, while for the test set, the RMSE and R^2^ were 6.726 and 0.836, respectively.

#### 3.2.3. Elastic Net (ENET)

ENET is a regularization technique that combines both LASSO and ridge regularization, with two hyperparameters to optimize: lambda (*λ*), which controls the overall strength of the penalty, and alpha (α), which determines the balance between the two types of penalties. When alpha is closer to 1, elastic net is similar to LASSO regularization, while when alpha approaches 0, it becomes closer to ridge regularization. Elastic net can generate reduced models by generating zero-valued coefficients, similar to LASSO regularization. The hyperparameter *α* allows the adjustment of the trade-off between feature selection (LASSO) and coefficient shrinkage (RIDGE).

The optimal combination for this model is a *λ* value of 0.029 and an *α* of 1, indicating that the model is similar to LASSO. The RMSE and R^2^ for the model were 4.041 and 0.937, respectively. On the training set, an RMSE of 2.898 and R^2^ of 0.966 were obtained, while on the test set, an R^2^ of 0.882 and an RMSE of 6.941 were obtained.

A comparison of nine machine learning techniques for quantitative determination of adulterant in honey using Fourier transform infrared spectroscopy with attenuated total reflectance (FTIR-ATR) found the ENET model was the best for determination of corn, cane, beet, and rice adulterants in honey. ENET also outperformed PLS, which is the traditional technique employed for multivariate regression in honey adulteration analysis [17].

#### 3.2.4. Partial Least Square (PLS)

PLS is a regression model that uses orthogonal principal components to optimize the explained power of response variables. It estimates regression coefficients for each latent variable and determines the optimal number of latent variables by minimizing the RMSE between predicted and observed response variables.

The final value of number of components used for the PLS model and determined by CV was 3, with an RMSE of 2.977 and an R^2^ of 0.972. On the training set, the RMSE was 2.882 and the R^2^ was 0.966. On the test set, the RMSE was 7.308 and the R^2^ was 0.875.

#### 3.2.5. Random Forest (RF)

RF combines multiple decision trees to improve the accuracy and robustness of the model. Each decision tree in the RF model is trained on a bootstrap sample of the original dataset, meaning that some data points are left out of the training process and used as out-of-bag (OOB) samples. RF randomly selects a subset of features before evaluating each split in an individual tree, which reduces the correlation between trees and prevents overfitting. The hyperparameter *mtry* determines the number of features randomly sampled at each split. The *mtry* parameter determines the size of this subset. It sets the maximum number of features that can be considered when deciding the best split at each node of a decision tree. A larger *mtry* value allows more features to be considered at each node. This can lead to more complex individual trees but can also lead to overfitting. Setting a smaller value of *mtry*, each tree is built using a limited set of features. This can enhance model interpretability because it leads to simpler trees and it is easier to understand the importance of specific features. However, it can result in a less diverse and potentially less powerful ensemble. The *mtry* value was chosen by trying different values and selecting the one that results in the best performance, using 5-fold CV. The best *mtry* value using a grid search method from 1 to 30 was 1, and the number of trees was established at 500. The number of trees in a random forest is an important hyperparameter, and it has a significant impact on the model’s performance. Increasing the number of trees generally improves the model’s accuracy. Random forests work by aggregating the predictions from multiple decision trees, and the ability of the model to generalize from the data tends to improve. This can lead to a reduction in both bias and variance, resulting in a more robust and accurate model. However, the computational cost increases significantly. In this case, the number of trees selected was a good balance between accuracy and computational cost.

The RMSE and R^2^ achieved by the model were 6.095 and 0.881, respectively. On the training set, an RMSE of 3.430 and R^2^ of 0.953 were obtained, while on the test set, an R^2^ of 0.869 and an RMSE of 6.285 were obtained.

#### 3.2.6. Support Vector Regression (SVR)

SVR is a supervised machine learning algorithm that uses a hyperplane to approximate a mapping function between the input variables and the output variables. To find the hyperplane that maximizes the margin between the closest points in the training set and the hyperplane, SVR uses two important parameters that have to be tuned: the cost of loss function (*C*) and kernel function (γ). The *C* parameter is the regularization parameter that controls the trade-off between model complexity and error. High *C* values make the model fit the training data more closely, potentially overfitting, while lower *C* values lead to a wider margin and less overfitting. The hyperparameter γ determines the width of the kernel and influences the decision boundary. Higher values of gamma result in a more localized and flexible kernel, while lower values lead to a smoother and more extended kernel. This adaptability allows the model to be fine-tuned and enables control over the trade-off between bias and variance. In this study, SVR was used with a radial basis function (RBF) kernel against a lineal kernel. The RBF kernel is highly effective at capturing non-linear relationships in the data. It allows SVR to model complex, non-linear patterns, which can be particularly beneficial when the relationship between the input features and the target variable is not adequately represented by a linear model.

Both hyperparameters (*C*, γ) were optimized by a grid search method using exponentially sequences from log_2_γ, log_2_C in a range of [−10, 10] every 0.5. The best results were obtained for a γ of 1.381× 10^−3^ and a C of 1024, which achieved an RMSE of 2.953 and an R^2^ of 0.973. On the training set, the RMSE was 2.700 and the R^2^ of 0.970, while for the test set, the RMSE was 6.336 and the R^2^ 0.909.

In summary, all models were evaluated as excellent in terms of effectiveness, achieving RPD values greater than 2.5. SVR outperformed other regression models in predicting the level of the adulterant in honey samples using a dataset containing OB and SF, considering the values achieved in all the metrics. Nevertheless, the results of the paired *t*-test showed no statistically significant difference (*p* > 0.05) between SVR, LASSO, and PLS using RSME.

The differences in the performances of the models can be attributed to the characteristics of the various machine learning algorithms. LASSO and RIDGE are regularization techniques that help to prevent overfitting by penalizing large coefficients but may struggle with non-linear patterns in complex datasets like honey composition. ENET combines LASSO and RIDGE penalties, balancing feature selection and coefficient shrinkage. However, it may still face challenges with highly non-linear data. PLS is useful for reducing dimensionality and handling multicollinearity, though it may not capture intricate non-linear relationships well. Although RF captures non-linear patterns and interactions by averaging decision trees, it can be computationally intensive and prone to overfitting with small datasets. In contrast, SVR is known for handling high-dimensional, complex data and can model non-linear relationships through kernel functions, which likely contributed to its superior performance in this study.

This result agrees with previously reported research using SVR applied to NIR spectroscopic data for the determination of adulteration in honeys [28]. Adulteration in honey can be influenced by various factors, and these relationships are often non-linear. In a study on the identification of the botanical origin and quantification of honey adulteration using Raman spectroscopy to compare PLS, SVR, and a convolutional neural network (CNN), the performance of the CNN models was significantly better than that of PLS and SVR. However, the predicted results of SVR were better than those of PLS [39].

### 3.3. Honey Botanical Origin Classification

An approach that can be used to improve the performance of regression models is to classify honey samples based on their botanical origin first, and then apply regression models separately. Discrimination of honey samples according to their botanical origin can help to ensure that the regression models are trained on data that are more homogeneous; the regression models can better capture the relationships between the different types of honey, and this can lead to more accurate predictions of the level of adulteration in honey samples.

#### 3.3.1. Principal Component Analysis (PCA)

**Figure 2:**
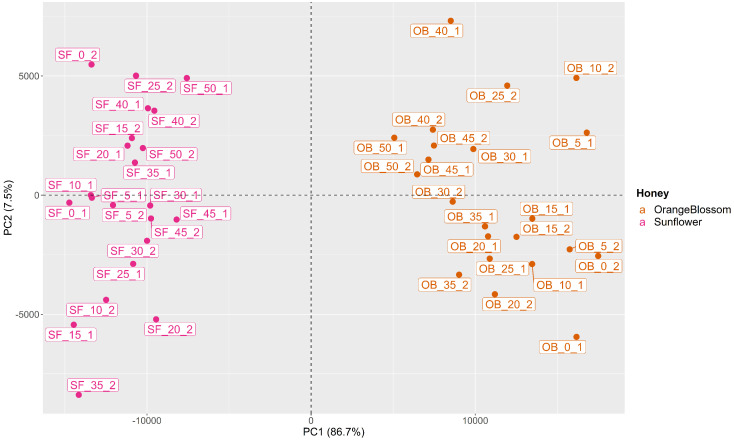
Score plot of PCA for orange blossom and sunflower honey.

PCA was performed to identify differences between OB and SF honey (*D*_44*x*20002_). Figure 2 shows the scores obtained by the samples for the first two principal components (PCs). The first principal component (PC1) and the second principal component (PC2) represented 86.7% and 7.5% of the cumulative variance, respectively, covering 94.2% of the total variance of the dataset. In the score graph (Figure 2), the samples of different botanical origin were distributed in two clearly differentiated areas based on their scores with respect to PC1 and PC2.

The first principal component (PC1) was found to be the most important in the explanation of the total variance in the dataset. Samples with positive scores on PC1 were associated with OB honey, while negative scores corresponded to SF honey. Additionally, the proximity of a sample’s PC1 value to 0 appears to be related to its adulteration level, with samples having higher levels of adulterants having values closer to 0. This trend is a consequence of the greater similarity among samples with higher adulterant levels, resulting in their proximity. The second principal component (PC2) did not provide significant additional information beyond PC1. It must be noted that PC2 contributes 7.5% of the total variance, whilst PC1 accounts for 86.7%. Other PCs were explored but did not provide additional insights into the data.

#### 3.3.2. Supervised Models for Classification According to Botanical Origin

PCA can be a useful first step in identifying patterns in data. In this case, it seems that the two groups are clearly separable based on the data obtained by UF-GC. However, to accurately classify new samples into one of the two groups, it is necessary to apply ML. SVM and RF are nonparametric techniques that can be used for building predictive models.

For classification purposes based on the botanic origin (OB and SF), the dataset (D_44*x*20002_) was randomly split into a 75% training set (*n* = 33) and a 25% test set (*n* = 11). Then, the training set was pre-processed in the same way as for the regression models, using the Boruta algorithm. In this case, 330 features were selected as relevant in the determination of the botanical origin of honeys, resulting in a reduced training dataset of D_33*x*330_. Model performance was also assessed by 5-fold-CV, and the metric used to evaluate the performance of the generated SVM and RF models was the accuracy, which was calculated as the number of correctly classified instances divided by the total number of instances.

A summary of the accuracy achieved by these models and the optimized parameters is shown in Table 4. As can be seen, both algorithms achieved an accuracy of 100% in the 5-fold CV, training set, and test set.

The results obtained through the RF and SVM models confirmed the applicability of these techniques for the discrimination of honey according to their botanical origin. In this study, other classification techniques, i.e., LASSO, RIDGE, and PLS, were explored but not included. They also obtained an accuracy of 100%.

A preview study demonstrated that a combination of isotope ratios and elements, along with a random forest algorithm, can effectively distinguish the botanical origin of Chinese honeys. The study found that the random forest model had a superior prediction accuracy of 96.5%, compared to other popular algorithms like SVM (91.5%), LDA (88.8%), and CART (82.1%). Overall, the random forest algorithm showed higher classification accuracy and greater robustness compared to the other typical algorithms [40].

#### 3.3.3. Prediction of Level of Adulterant in Orange Blossom and Sunflower Honey

Once the floral origin was identified, the next step was to establish the level of adulteration in each type of honey. For this, the Boruta variable selection algorithm was applied to the dataset belonging to each type of honey. In the case of OB honey, 50 variables (D_22*x*50_) were selected, and 59 variables were selected for SF honey (D_22*x*59_). Finally, the same regression algorithms previously used for the dataset containing all the honey samples were evaluated. Hyperparameter tuning, using grid search, random search, or Bayesian optimization, is crucial for finding the best hyperparameters to optimize model performance. In this case, a grid search strategy was selected. A summary of the optimized parameters, the performance of the regression models, and the performance in the training and test sets applied to all honey samples, and only to OB and SF honey, is shown in Table 2 and Table 3.

It should be noted that only the SVM regression model was previously selected as the best model for all honey samples, with an RMSE of 6.336 and an R^2^ of 0.909, since the other models provided R^2^ values less than 0.9. However, when the honey samples were treated separately, the performance of all the regression models improved, obtaining values of R^2^ greater than 0.95. These results suggest that treating OB and SF honey samples separately can lead to improved model performance compared to using all the honey samples together.

The results also suggest that all the regression models tested in OB and SF honey separately obtained a similar performance, with an R^2^ in the test set higher than 0.990, except for the RIDGE model in OB honey (0.9843). In the case of RMSE in the test set, LASSO was found to be the best model for predicting the properties of both OB and SF honey, obtaining RSME values of 1.306 and 1.357, respectively. This highlights the importance of considering different regression models and selecting the one that performs best for a specific dataset. However, the paired *t*-test indicated no statistically significant differences (*p* > 0.05) regarding RSME in all the ML models.

This highlights the importance of considering different regression models and selecting the one that performs best for a specific dataset. Also, the performance of each machine learning technique greatly depends on the selection and values of its parameters, and it is important to note that the parameter selection can be a limitation of machine learning algorithms, as suboptimal parameter choices may lead to poor model performance. We implemented a grid search method to address this limitation in our ML models. Another approach to enhance the performance of this methodology is to use stacking ensembles, which integrate predictions from multiple ML algorithms for potentially improved accuracy. However, implementing stacking can be complex. In a previous study [17], the effectiveness of a stacked ensemble approach was tested, but it did not outperform the ENET regression technique. Probably, more research concerning stacking ensembles is still needed.

## 4. Conclusions

This research highlights the potential of machine learning models, particularly support vector regression (SVR) and LASSO, in enhancing the detection of the adulteration of higher-value honey with lower-value honey. This study developed predictive models, specifically a support vector regression (SVR) model, which achieved R^2^ values above 0.90 on a combined dataset of orange blossom (OB) and sunflower (SF) honey. Treating OB and SF honey separately further improved model performance, reaching R^2^ values over 0.99. Among the models, LASSO showed strong potential for predicting adulteration when applied to each honey type individually. These methods may significantly enhance adulteration detection and serve as a screening tool for local labs and food safety authorities.

## Figures and Tables

**Table 1:** Summary of limitations and challenges in major techniques used for detecting honey adulterations.

Technique	Principle/Compounds Involved	Limitations and Challenges
Stable Carbon Isotope Ratio Analysis (SCIRA)	Isotopic ratios of sugars	Limited resolution, expensive equipment
Gas Chromatography (GC)	Analysis of volatile compounds	Miss non-volatile adulterants, complex sample preparation
Ion Mobility–Mass Spectrometry (IMS)	Analysis of volatile compounds	Limited resolution, quantification challenges
Nuclear Magnetic Resonance (NMR)	Joint spectra mainly from sugars	Limited sensitivity, high cost, skill-dependent
Fourier Infrared Spectroscopy with ATR (FTIR-ATR)	Joint spectra mainly from sugars	Limited specificity, vulnerable to impurities
UV–Visible (UV-Vis) and Near-Infrared (NIR) Spectroscopy	Joint spectra mainly from sugars	Lack of specificity, need for comprehensive databases
Raman Spectroscopy	Joint spectra mainly from sugars	Limited penetration, sensitivity to sample properties
Thermographic Images	Surface temperature mapping	Limited applicability, interpretation challenges

**Table 2:** Optimized parameters and 5-fold CV performance of regression models applied to all honey samples and to OB and SF honeys. LASSO = least absolute shrinkage and selection operator; RIDGE = ridge regression; ENET = elastic net regression; PLS = partial least squares regression; LVs = latent variables; RF = random forest; SVR = support vector regression with radial kernel; ALL = containing all botanical origin honey samples; OB = orange blossom honey; SF = sunflower honey.

Algorithm	Dataset	Hyperparameters	5-Fold CV Performance	
			RMSE	R^2^
LASSO	All	*λ* = 0.132	4.041	0.937
	OB	*λ* = 0.305	2.340	0.990
	SF	*λ* = 0.201	2.712	0.992
RIDGE	All	*λ* = 10	6.353	0.901
	OB	*λ* = 10	3.508	0.973
	SF	*λ* = 10	3.404	0.991
ENET	All	*α* = 0.03 *λ* = 1	4.041	0.937
	OB	*α* = 0.1 *λ* = 0.923	2.297	0.989
	SF	*α* = 0.1 *λ* = 0.100	2.631	0.993
PLS	All	3 LVs	2.978	0.972
	OB	3 LVs	2.112	0.996
	SF	3 LVs	2.710	0.995
RF	All	*Mtry* = 1	6.095	0.881
	OB	*Mtry* = 46	4.452	0.986
	SF	*Mtry* = 40	3.399	0.980
SVR	All	γ = 1.381 × 10^−3^ *C* = 1024	2.953	0.973
	OB	γ = 9.766 × 10^−4^ *C* = 32	3.099	0.989
	SF	γ = 3.906 × 10^−3^ *C* = 45.255	2.036	0.992

**Table 3:** Performance of regression models applied to all honey samples and to OB and SF honeys on the training and test set. LASSO = least absolute shrinkage and selection operator; RIDGE = ridge regression; ENET = elastic net regression; PLS = partial least squares regression; RF = random forest; VM = support vector regression with radial kernel; ALL = containing all botanical origin honey samples; OB = orange blossom honey; SF = sunflower honey. Subscripts refer to significance between models assessed by paired *t*-test.

Algorithm	Dataset	Training Set Performance (*n* = 33)			Test Set Performance (*n* = 11)		
		RMSE	R^2^	RPD	RMSE	R^2^	RPD
LASSO	All_A_	3.745	0.943	4.235	6.335	0.874	2.707
	OB	1.615	0.990	10.507	1.306	0.994	16.120
	SF	1.956	0.985	8.365	1.357	0.999	12.667
RIDGE	All_B_	5.480	0.877	2.894	6.726	0.836	2.550
	OB	3.737	0.977	4.468	5.026	0.984	4.181
	SF	3.293	0.979	4.999	2.300	0.998	7.572
ENET	All_A_	2.898	0.966	5.473	6.941	0.882	2.471
	OB	1.603	0.990	9.944	1.365	0.994	14.340
	SF	3.668	0.948	8.058	1.399	0.999	12.295
PLS	All_A_	2.883	0.966	0.714	7.308	0.875	2.347
	OB	1.737	0.987	9.534	1.732	0.992	13.273
	SF	2.159	0.982	7.629	1.518	0.999	11.420
RF	All_C_	3.430	0.953	4.623	6.284	0.868	2.729
	OB	4.666	0.988	7.789	5.112	0.964	3.976
	SF	1.983	0.989	8.752	2.100	0.996	7.785
SVR	All_A_	2.700	0.970	5.872	6.336	0.909	2.706
	OB	1.639	0.990	9.743	1.435	0.995	12.19
	SF	1.452	0.992	11.516	1.928	0.999	9.359

**Table 4:** Summary of botanical origin classification models on honey samples. RF = random forest; SVM = support vector regression with radial kernel.

Algorithm	Hyperparameters	5-Fold CV Accuracy (%)	Training Set (*n* = 33) Accuracy (%)	Test Set (*n* = 11) Accuracy (%)
RF	*mtry* = 19 γ = 4.883 × 10^−4^	100	100	100
SVM	C = 0.5	100	100	100

## Data Availability

Data are contained within the article.
